# Visual Fixations and Motion Sensitivity: Protocol for an Exploratory Study

**DOI:** 10.2196/16805

**Published:** 2020-07-27

**Authors:** Shikha Chaudhary, Nicola Saywell, Arun Kumar, Denise Taylor

**Affiliations:** 1 Auckland University of Technology Auckland New Zealand; 2 Manipal Institute of Technology Manipal, Karnataka India

**Keywords:** motion sensitivity, vestibular disorder, complex environments, visual fixations, postural control, posture, kinematics, inner ear, visual

## Abstract

**Background:**

Motion sensitivity after vestibular disorders is associated with symptoms of nausea, dizziness, and imbalance in busy environments. Dizziness and imbalance are reported in places such as supermarkets and shopping malls which have unstable visual backgrounds; however, the mechanism of motion sensitivity is poorly understood.

**Objective:**

The main aim of this exploratory observational study is to investigate visual fixations and postural sway in response to increasingly complex visual environments in healthy adults and adults with motion sensitivity.

**Methods:**

A total of 20 healthy adults and 20 adults with motion sensitivity will be recruited for this study. Visual fixations, postural sway, and body kinematics will be measured with a mobile eye tracker device, force plate, and 3D motion capture system, respectively. Participants will be exposed to experimental tasks requiring visual fixation on letters, projected on a range of backgrounds on a large screen during quiet stance. Descriptive statistics (mean and standard deviation) will be calculated for each of the variables. One-way independent-measures analyses of variance will be performed to investigate the differences between groups for all variables.

**Results:**

Data collection was started in May 2019 and was completed by February 2020. It was approved by Health and Disability Ethics Committees, Ministry of Health, New Zealand on November 2, 2018 (Ethics ref: 18/CEN/193). We are currently processing the data and will begin data analysis in July 2020. We expect the results to be available for publication by the end of 2020. The trial was funded by the Neurology Special Interest Group, Physiotherapy New Zealand, and the Eisdell Moore Centre in November 2018.

**Conclusions:**

This study will provide a detailed investigation of visual fixations in response to increasingly complex visual environments. Investigating characteristics of visual fixations in healthy adults and those with motion sensitivity will provide insight into this disabling condition and may inform the development of new intervention strategies which explicitly cater to the needs of this population.

**Trial Registration:**

Australian New Zealand Clinical Trials Registry, ACTRN12619000254190; https://tinyurl.com/yxbn7nks

**International Registered Report Identifier (IRRID):**

PRR1-10.2196/16805

## Introduction

Motion sensitivity is characterized by nausea, dizziness, and imbalance in response to motion of the visual environment [1]. It can develop as a sequela of a vestibular disorder and is one of the diagnostic criteria for persistent postural perceptual dizziness [1-3]. The symptoms are due to a misinterpretation of, or overreliance on, visual cues for orientation in space [1,3-6]. Dizziness and imbalance are triggered in busy surroundings with visual motion or complex repetitive patterns. Consequently, people with motion sensitivity tend to avoid crowded or busy environments such as supermarkets or driving on motorways [7]. This frequently leads to an interruption of daily activities, sick leave from work, and in extreme cases a reluctance to leaving the house [8,9]. Motion sensitivity may affect people following an acute vestibular insult or people with chronic recurrent dizziness [10].

**Figure 1:**
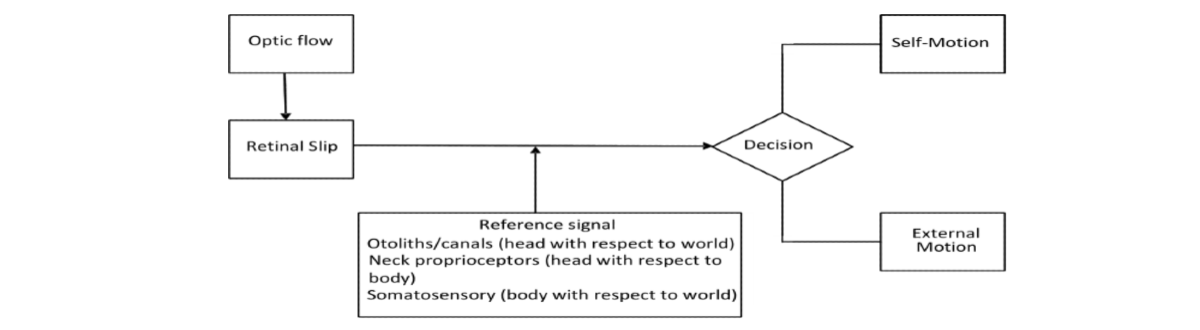
The sources of information to allow differentiation of self-motion and object motion components. The various sources are shown as giving information with respect to different reference frames.

Information from the visual system has a role in differentiating self-motion from external motion [11]. This differentiation is dependent on perceiving whether motion on the retina is due to an object moving relative to the person or the person moving relative to the object [12,13]. This distinction between self-motion and external motion is achieved by a mechanism that compares the retinal signal and the reference signal. The reference signal comprises information from vision, vestibular afferents, proprioceptive feedback from the extraocular muscles, somatosensory kinaesthetic proprioception, and cognition [14] (Figure 1).

A crucial aspect of the stabilization of posture is dependent on the visual input received from the environment. An essential component of visual input is optic flow [15,16]. Optic flow helps perception of spatiotemporal information from the environment which is then used to move around and maintain orientation in space [12,17]. Optic flow generates retinal slip, defined as motion of the visual image of the environment on the surface of the retina [18]. This information is used to adjust the amount of postural sway. The main aim of visually induced postural movements is to reduce the overall amplitude of the optic flow field by minimizing retinal slip [19-21].

Because optic flow plays a vital role in postural correction, perceiving inaccurate information can be destabilizing. Optic flow that is a part of the background motion behind a target is not normally used as a visual input for postural control as it can stimulate an optokinetic response (which evokes a combination of a slow-phase and fast-phase eye movements where the eyes momentarily follow the moving object and then rapidly reset back to the initial position) [22]. This response can induce a standing subject to move in response to the direction of the motion and can be destabilizing [22]. In normal circumstances, this optokinetic response to background motion is suppressed by visually fixating on a target [23].

Visual fixations contribute to 80% of the total visual experience [24], and help to reduce optic flow, minimize retinal slip, and suppress the optokinetic response [23]. When fixating on a stationary target, there is almost no retinal slip, and the vestibulo-ocular reflex keeps the gaze on target during head movements [21]. By contrast, maintaining fixation on a moving object requires suppression of the vestibulo-ocular reflex for the eyes and the head to move in the same direction [21,25,26].

Fixations contribute to a person’s sense of spatial orientation. Fixations suppress visual field motion perception by maximizing the peripheral vision and rendering a stable image to enhance the visual signals of self-motion [27]. Sensations of small body movements then facilitate the execution of compensatory postural reactions [28].

Fixational instability may predispose a person to develop motion sensitivity [29-31]. Studies have shown that people with motion sensitivity after vestibular disorders exhibit fixational instability and have increased perceptual and postural responses to complex visual surroundings [31-34]. Several studies have investigated the relationship between fixational instability and the strength of illusionary motion [29-31]. Fixational instability can be detected by frequency of refixations and saccades [32,34]. Studies have reported that a person with fixational instability would have a high frequency of saccades and refixations while attempting fixation [32,34].

Any difficulty in differentiating self-motion from external motion will require adjustments to determine the correct orientation in space. A peripheral or central vestibular disorder disrupts the normal visual–vestibular interaction [35], which can alter the perception of motion. It can lead to illusionary motion perception, thereby degrading postural stability. Adults with motion sensitivity report worsening of symptoms and reduced postural control in visually stimulating environments which may be explained by fixation instability. However, to date, visual fixations have not been well investigated in people with motion sensitivity. Previous studies have used video oculography or electrooculogram and optokinetic stimulation rotating around the naso-occipital center to study eye movements in adults with motion sensitivity [32-34]. This study aims to investigate the characteristics of fixations in people with motion sensitivity and how they differ from those of healthy adults by using a mobile eye tracker device in a more naturalistic yet controlled laboratory setting.

This research will investigate visual fixations, postural sway, and kinematics of adults with motion sensitivity, compared with healthy adults, in complex visual environments. Center of pressure (COP) measurement will be used to evaluate postural sway. COP parameters have been used widely to describe stability and quantify alterations in postural control [36,37]. The exploratory nature of this study will also allow the investigation of mean saccadic velocity and saccadic peak amplitude between groups. Several studies have identified anomalies in mean saccadic velocities in a range of health conditions [38,39].

This study is the first step toward recognizing the components that may be essential in a rehabilitation programme addressing the challenging clinical issue of motion sensitivity and may guide the development of rehabilitation programs for adults with motion sensitivity.

## Methods

### Aim

To conduct an observational study with 40 adults (20 in each group; healthy adults and adults with motion sensitivity). The study will determine whether complex visual environments are associated with fixational instability, altered COP displacement, and altered center of mass (COM) displacements of the head and body in adults with motion sensitivity compared with healthy adults.

### Hypothesis

Complex visual environments in people with motion sensitivity compared with healthy adults will be associated with (1) increased number of visual refixations, (2) increased displacement of COP, and (3) differences in the body COM displacement and differences in the head COM displacement.

### Trial Design, Setting, and Participants

This is a cross-sectional exploratory single-session experimental study that will be laboratory based in Auckland University of Technology. A total of 40 adults will participate in the study (20 healthy adults and 20 adults with motion sensitivity after vestibular disorder). Healthy adults aged between 18 and 60 who are independently mobile and have no history of neurological conditions will be recruited through neurorehabilitation research team networks and community advertisements. Adults with motion sensitivity will be recruited through a specialized vestibular disorders clinic. They will be included if they have had a history of vestibular disorder (confirmed by a clinician in the vestibular disorder clinic) but have no current signs of acute vestibular deficits, are aged between 18 and 60 [40,41], have a history of motion sensitivity symptoms as reported by the Visual Vertigo Analogue Scale (score >5) [42-44], and score >40 on the Dizziness Handicap Inventory [44]. People with a history of previous eye surgery, or a medical condition that may influence eye movements such as sarcoidosis, Lyme disease, diabetes mellitus, traumatic brain injury, and migraine will be excluded from the study.

### Recruitment

Potential participants will be provided with a Participant Information Sheet and be requested to contact the corresponding author by email or telephone. All potential participants will be made aware that participating in this study will not influence their current health care.

### Screening

Potential participants will be screened against the study’s inclusion and exclusion criteria via telephone or through a face-to-face meeting with the researcher (SC). Eligible potential participants will be asked to provide written informed consent.

### Experimental Setup

**Figure 2:**
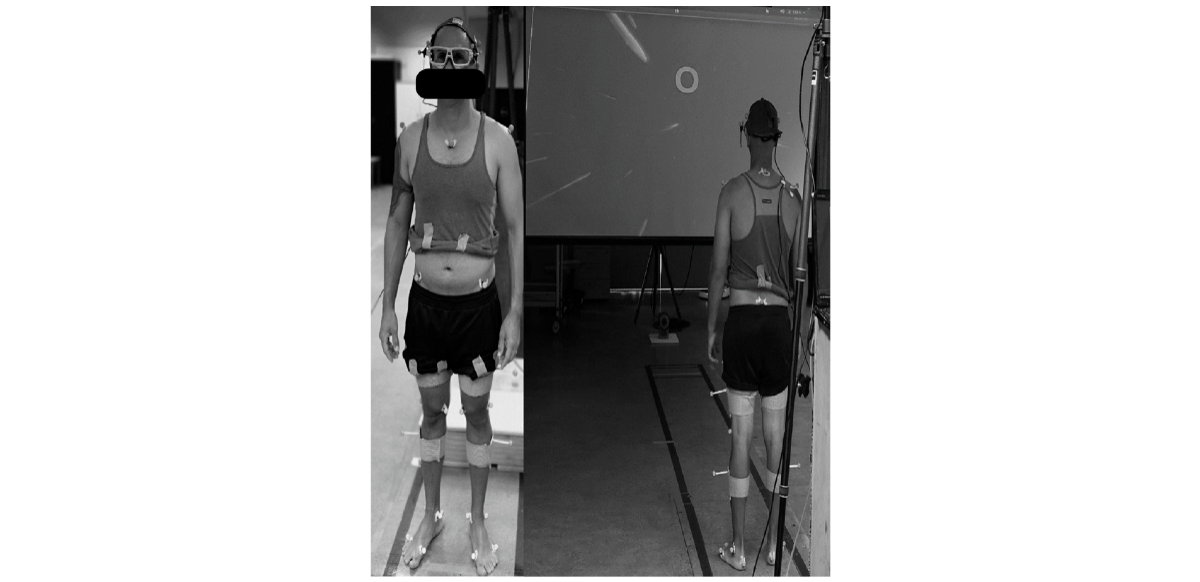
The experimental setup.

The experimental setup consists of a projector screen (Brateck Lumi), a mobile eye tracker device (SensoMotoric Instruments), a force plate (Advanced Mechanical Technology Inc.), and a 3D motion capture system (Qualisys Motion Analysis Capture System; Qualisys Medical AB). Visual fixations will be recorded using a mobile eye tracker (SMI BeGaze; SensoMotoric Instruments). A 3D motion capture system and a force plate will be used to record kinematics and postural sway, respectively. The projector screen (135 in., 16:9 aspect ratio) will be mounted at 3.5-m distance from the force plate for projecting the visual environments (Figure 2).

#### Sensor Motoric Instruments Eye Tracking Glasses (SMI ETG)

**Figure 3:**
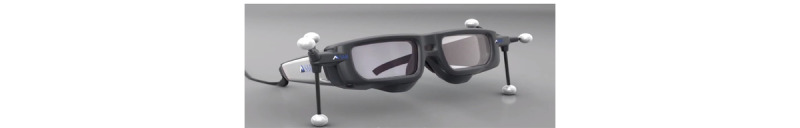
SMI Eye Tracking Glasses with 3D reflective markers.

The SMI Eye Tracking Glasses (SMI ETG) are a mobile eye-tracking device with a binocular sampling rate of 120 Hz (Figure 3). SMI uses infrared light of wavelength around 789-880 nm to increase the contrast between the pupil and iris which is easily detected by the camera. SMI is a video-based eye tracker based on the concept of pupil center corneal reflection. The scene camera has a resolution of 1280 × 960 pixels @ 24FPS, 960 × 720 pixels @ 30 FPS with a 60° horizontal and 46° vertical field of view. The gaze position accuracy is 0.5° for all distances and the gaze tracking range is 80° horizontal and 60° vertical.

The SMI software uses a frame-by-frame analysis of the gaze data. These data involve defining the location and type of gaze behavior for each frame of data collected. Frame numbers are used to determine the duration of the eye movement. SMI uses an in-built detector for identifying saccades, fixations, and blinks. According to the detector, a blink is identified by points where eye data are not present, a saccade represents a quick change in gaze location, and a fixation is bordered by 2 saccades.

Data collected by the eye-tracking glasses identify the primary event as fixation and therefore a dispersion-based algorithm is used. The algorithm identifies fixations as groups of successive points within a dispersion, or maximum separation. A blink is determined based on the whole trial data where the pupil diameter is either zero, or the horizontal and vertical gaze positions are zero, or they lie outside a calculated valid pupil range. Once fixations and blinks are identified, a saccadic event is created between the detected blinks and fixations.

#### Qualisys System

Qualisys is a motion capture system used to track movement. Small retro-reflective markers reflecting infrared light are attached to the participant’s skin. Frame-by-frame analysis is used to track each marker from one frame to the next. Each marker data and their 3D position trajectory can be used to calculate joint and movement trajectories. The force plates and SMI eye tracker are integrated and time synced in the Qualisys system. The SMI program is installed on the Qualisys system. The data from the SMI system are synchronized to Qualisys via a start command, so as to capture its data together with all the other data in the Qualisys system. The force plates are connected to the Qualisys computer via an analog board to capture analog signals from the force plate with the motion capture data. The data from the force plates are synchronized with the motion capture data via a synchronization signal between the Qualisys camera system and the analog board. The sync signal from the camera system is connected to an external trigger input on the analog board to start the capture of analog data using hardware synchronization.

Kinematics will be measured using an infrared motion analysis capture system, consisting of 9 Oqus 3D Motion analysis capture units. A set of 27 reflective markers will be placed on the participant. Markers will be attached using a double-sided tape directly onto the skin. Pelvis markers will be placed in accordance with the modified Helen Hayes model as a set of 3: 1 marker on each anterior superior iliac spine and 1 on sacrum (midpoint). For the thigh segment, markers will be placed on midthigh, medial femur epicondyle, and femur lateral epicondyle for each extremity. The shank segment includes markers on midshank, medial malleolus, and lateral malleolus. The foot segment will be created using a set of markers on the head of the fifth metatarsus, head of the first metatarsus, and posterior surface of the calcaneus. Further, markers will be placed on the right and left acromion process, sternoclavicular notch, and C7 vertebra to create the thorax segment. To create the head segment, 1 marker will be placed on each side of the head.

3D co-ordinates of each reflective marker will be tracked using Qualisys Track Manager. Visual 3D software will be used to process the data files. After placement of markers, a static image of the participant standing in an anatomical position will be taken.

#### AMTI Force Plates

Postural control as the COP movement will be measured with an AMTI force platform (Advanced Mechanical Technology Inc.). The force platform measures the 3 force components, *F_x_*, *F_y_*, and *F_z_* (where *x*, *y*, and *z* are the medial–lateral, anterior–posterior, and vertical directions, respectively), at the sampling frequency of 1200 Hz. The AMTI force plate is a static-force measurement system and is a computer-based system which synchronizes with a computer using a serial link. The COP movement track data (in millimeter) will be collected for each participant and will be converted into mediolateral and anterior–posterior components for analysis.

Participants will stand on the force plate with arms relaxed at their sides. Participants will be asked to stand with their feet shoulder width apart. They will be instructed to maintain their gaze on the letter while maintaining a quiet stance for the duration of a task.

### Experimental Tasks

**Figure 4:**
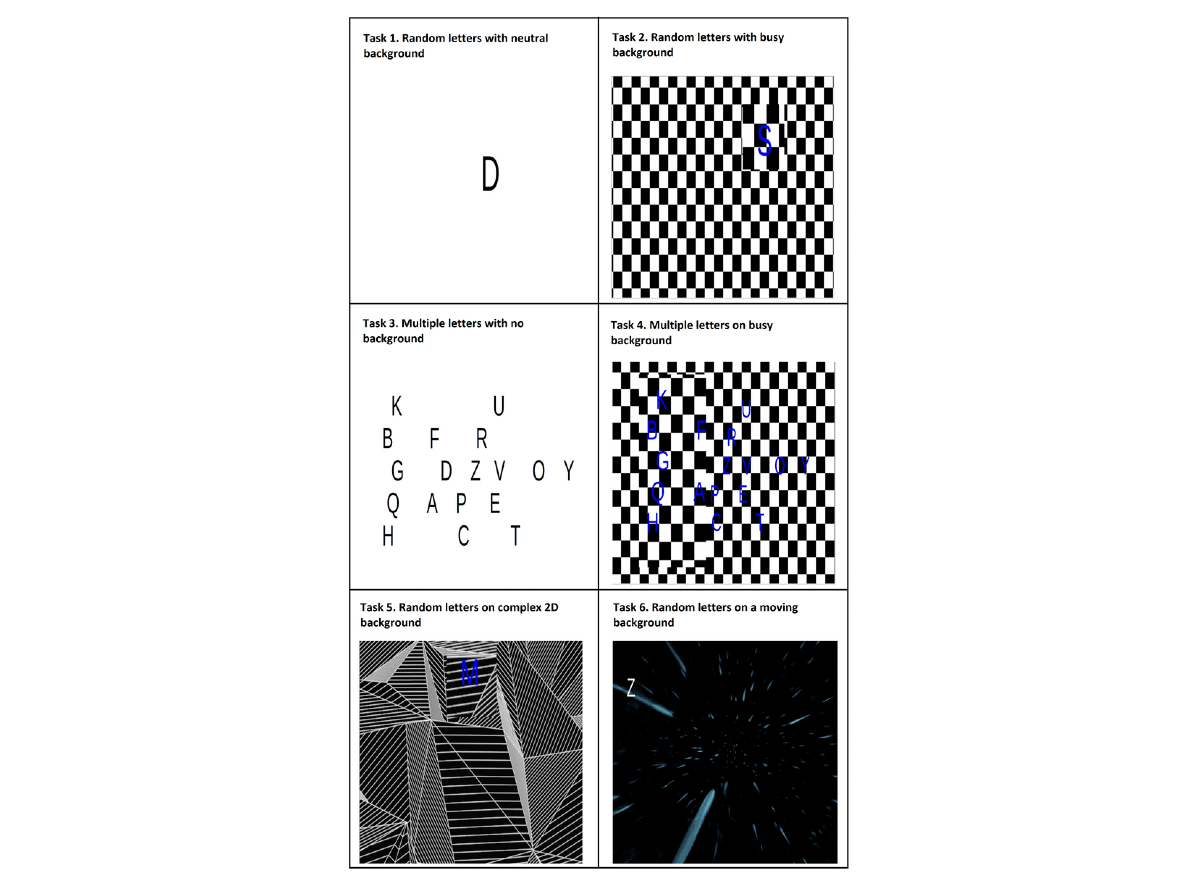
The experimental tasks. *Letters have been magnified for clear visibility.

The experimental tasks have been designed to simulate eye movements in visually complex environments. Tasks will increase in the level of complexity, starting from easy visual tasks progressing to more visually complex tasks. Letters will be projected in a random sequence on to a range of visually complex background images (Figure 4). The font of the letters, backgrounds, and duration of each task were finalized after piloting. There are 6 tasks, each lasting 70 seconds. The letters appear on the screen for 7 seconds each, at different positions on the screen. Python programming language has been used to select letters and their positions on screen. Participants will be instructed to focus on a letter as they appear on the screen. The tasks increase in difficulty in two ways: (1) the background behind the letter progresses from neutral to busy (ie, to a complex moving background), and (2) by the appearance of either a single letter or multiple letters on screen. In the single-letter tasks (tasks 1, 2, 5, and 6), the participants will be instructed to focus their gaze on each projected letter for the duration of the task. In the multiple letters’ tasks (tasks 3 and 4), the participant will be instructed to find letter *E* and maintain visual fixation on it for the duration of the task. The tasks will be presented from the lowest to highest difficulty of background and number of letters (as described in Figure 4). The tasks will not be randomized as the more difficult tasks might provoke symptoms of dizziness which would hinder the performance of participants in the subsequent tasks.

### Data Collection

**Figure 5:**
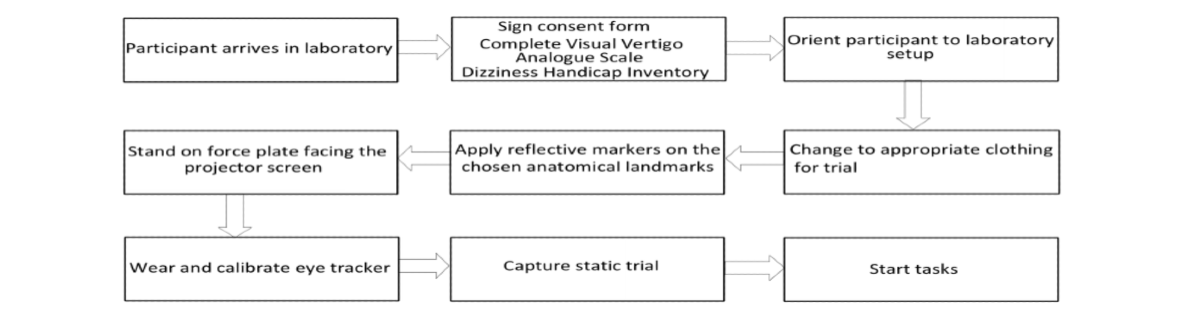
Data collection procedure.

Data will be collected for all tasks in 1 session. The motion analysis system and force plate will be calibrated before the participant arrives in the laboratory. Upon arrival, the participant will be orientated to the laboratory setup. The Dizziness Handicap Inventory and Visual Vertigo Analogue Scale screening will be completed. After setting up the markers, experimental tasks will be explained, and the eye tracker glasses will be fitted for comfort and calibrated. The participant will then stand on the force plate wearing the calibrated eye tracker with reflective markers (Figure 5). During the experimental tasks, appropriate rest intervals will be provided after each task to minimize provocation of symptoms such as dizziness and nausea.

### Outcome Measures

The following outcome measures will be explored and analyzed for this study.

#### Visual Fixations

Fixation characteristics for each group will be computed using the SMI ETG software and will measure the total number of refixations, the maximum fixation duration, and the number of saccades. The software determines a fixation as a window with a minimum duration of 80 ms and a maximum dispersion of 100 pixels. Refixations are calculated if the eye crosses the maximum dispersion threshold of 100 pixels. The fixation duration will be calculated as the total time spent in fixating during a trial. The maximum fixation duration will be calculated as the longest fixation within each trial. A saccade event is computed as any event that does not meet the fixation criteria between the new and the previous fixation.

#### Postural Sway: Center of Pressure

COP displacement times series obtained from the force platform will be down sampled to 100 Hz and subsequently will be processed using a low-pass filter at 5 Hz (fourth-order, zero-phase-lag, Butterworth) [45]. The mean velocity, root mean square, and maximum range of the COP displacement will be computed to evaluate postural sway [46].

#### Kinematics

Raw data from the Qualisys motion analysis will be imported into Visual 3D, where a 6 degree-of-freedom model will be constructed. Data will be interpolated and processed using a fourth-order Butterworth low-pass filter with cutoff frequency of 12 Hz. The body COM and the COM of the head segment will be calculated using a pipeline in Visual 3D. The mean velocity, root mean square, and maximum range of the COM of the head and whole-body displacement will be calculated [47].

### Safety Measures

The study will be using moving background/moving images, which may induce dizziness, imbalance, or nausea in some participants. An assistant will stand close to the participant to provide assistance and prevent a fall in case of imbalance. We will monitor how a participant is feeling throughout each task, and appropriate rest intervals will be provided. The session would be stopped at any stage if required.

In the unlikely event of a physical injury, rehabilitation and compensation for injury by accident may be available from the Accident Compensation Corporation, provided the incident details satisfy the requirements of the law and the Corporation’s regulations.

### Statistical Analysis

#### Sample Size Calculation

There is a lack of experimental evidence in the population of interest to conduct a power calculation for the required sample size. It is unclear if factors such as age or gender affect visual fixations and there is minimal information on population variation. Therefore, an arbitrary sample size of 20 in each group has been selected. This has been selected in accordance with studies performed in adults with motion sensitivity [33,34]. The data from this study may help inform future studies for the required sample size.

#### Analysis

Descriptive statistics (mean and standard deviation) will be calculated for each of the variables. Data normality will be examined using the Kolmogorov–Smirnov statistic. One-way independent-measures analyses of variance will be performed to investigate the differences between groups for all variables. Post-hoc analysis with Šidák adjustment will be used for multiple comparisons [48]. Finally, a receiver operating characteristic curve analysis will be applied to determine threshold values in gaze, COP, and COM parameters, allowing identification of the impairment induced by motion sensitivity. The optimal cutoff point will be determined using the Youden Index. Areas under the curve, specificity, and sensitivity will also be calculated. Values of areas under the curve will be categorized as follows: excellent (≥0.90), good (0.80-0.90), fair (0.70-0.79), and poor (<0.70).

### Confidentiality

During the screening, the researcher will make note of whether the potential participant meets the study criteria. For those who do not meet the criteria, only the reason for exclusion from the project will be recorded in a database and will not be identifiable.

### Ethics Approval and Consent to Participate

Ethical approval for this study has been obtained from the New Zealand Health and Disability Ethics Committee (HDEC) and Auckland University of Technology Ethics Committee (AUTEC). Eligible potential participants will be asked to provide written informed consent. Ethics committee approval for any protocol modifications will be sought from HDEC and AUTEC. Any changes will lead to an amendment in the Australian New Zealand Clinical Trials Registry (HDEC reference number: 18/CEN/193; AUTEC reference number: 19/38).

### Dissemination of Study Data

A summary of the results from the study will be offered to all participants as per the consent form. Results from the study will be published in a peer-reviewed journal and presented at national and international conferences.

### Availability of Data and Materials

All participants will be given a numerical code upon acceptance into the project. All health information will be stored in physical and electronic records that are identified by the participant code only. Only the named investigators will have access to the forms that contain information about the participant’s name and their code. These forms will be stored in a secured cabinet in co-ordinating investigator’s office, separate from any records containing health information.

The data sets used and analyzed during this study are available from the corresponding author on reasonable request.

## Results

Data collection was started in May 2019 and was completed by February 2020. It was approved by the Institutional Review Board on November 2, 2018 (Ethics ref: 18/CEN/193). We are currently processing the data and will begin data analysis in July 2020. We expect the results to be available for publication by the end of 2020. The trial was funded by the Neurology Special Interest Group, Physiotherapy New Zealand, and the Eisdell Moore Centre in November 2018.

## Discussion

This is an exploratory study with the primary aim to identify whether fixational instability is associated with motion sensitivity and whether it leads to increased postural sway and altered kinematics in adults with motion sensitivity.

This study will provide a detailed investigation of visual fixations, postural sway, and kinematics in complex visual environments. The use of a mobile eye tracker device will investigate naturalistic eye behavior when exposed to experimental stimuli. The task hierarchy will help in understanding how characteristics of visual fixations change when a person views a complex visual environment as opposed to neutral environments. The experimental tasks might provoke symptoms in some participants; however, we expect that all participants will be able to complete the protocol with appropriate rest intervals between tasks. Our sample size of 20 participants in each group is a foundational step in exploring whether visual fixations contribute to motion sensitivity after vestibular disorder. We anticipate the outcomes will be able to detect a difference between healthy adults and those with motion sensitivity. Results from this study will inform future trials and will be used to inform development of diagnostic and rehabilitation programs.

We hope that this study will increase our understanding of the complex interactions of vision and balance in people with motion sensitivity. If we determine that gaze and postural control characteristics are altered, we will develop an intervention that is designed to re-align the gaze and postural control characteristics closer to those of the control population. This intervention would then be tested in a series of clinical trials to determine effectiveness.
